# An Ultra-Low Power CMOS Image Sensor with On-Chip Energy Harvesting and Power Management Capability

**DOI:** 10.3390/s150305531

**Published:** 2015-03-06

**Authors:** Ismail Cevik, Xiwei Huang, Hao Yu, Mei Yan, Suat U. Ay

**Affiliations:** 1 Department of Electrical and Computer Engineering, University Idaho, 875 Perimeter Drive MS 1023 Moscow, ID 83844-1023, USA; E-Mail: ismailc@uidaho.edu; 2 School of Electronics and Information, Hangzhou Dianzi University, No.1 Avenue, 2 Xiasha, Hangzhou 310018, China; E-Mail: gfhuangxiwei@gmail.com; 3 Electrical and Electronic Engineering Department, Nanyang Technological University (NTU), 50 Nanyang Avenue, 639798, Singapore; E-Mails: haoyu@ntu.edu.sg (H.Y.); yanmei@ntu.edu.sg (M.Y.)

**Keywords:** ultra-low power, CMOS image sensor, energy harvesting, power management, MPPT

## Abstract

An ultra-low power CMOS image sensor with on-chip energy harvesting and power management capability is introduced in this paper. The photodiode pixel array can not only capture images but also harvest solar energy. As such, the CMOS image sensor chip is able to switch between imaging and harvesting modes towards self-power operation. Moreover, an on-chip maximum power point tracking (MPPT)-based power management system (PMS) is designed for the dual-mode image sensor to further improve the energy efficiency. A new isolated P-well energy harvesting and imaging (EHI) pixel with very high fill factor is introduced. Several ultra-low power design techniques such as reset and select boosting techniques have been utilized to maintain a wide pixel dynamic range. The chip was designed and fabricated in a 1.8 V, 1P6M 0.18 µm CMOS process. Total power consumption of the imager is 6.53 µW for a 96 × 96 pixel array with 1 V supply and 5 fps frame rate. Up to 30 μW of power could be generated by the new EHI pixels. The PMS is capable of providing 3× the power required during imaging mode with 50% efficiency allowing energy autonomous operation with a 72.5% duty cycle.

## 1. Introduction

Recently, CMOS image sensors (CISs) have replaced charge-coupled devices (CCD) in most applications due to their low power consumption, high speed, and low cost. Since CISs are built using the same CMOS manufacturing processes used for building analog and digital integrated circuits (ICs), they are suitable for system-on-chip (SOC) integration. CIS SOCs integrate readout electronics, digital control, timing, and image processing circuits and other system components on the same die to build compact imaging systems. In addition, the introduction of backside illumination (BSI) has improved the pixel fill factor and quantum efficiency of CIS pixels that in turn has improved low-light performance, surpassing CCD quality. 

Today, low-power consumption is a critical requirement that enables standalone operation of sensors and systems in isolated environments for extended durations. This becomes especially critical for implantable medical systems [1,2,3]. A system capable of harvesting ambient energy in the environment can achieve significantly longer operating lifetime. Integrating an energy harvester on the same silicon die to power up the system can avoid many unnecessary energy loss paths while conserving available energy. Since most CISs operate in illuminated environments, photovoltaic (PV) energy harvesting is the natural choice among all the different kinds of energy harvesters. PV energy harvesters have relatively high conversion efficiency and they are compatible with standard CMOS processes as well [4,5].

A CIS can achieve energy autonomous operation by harvesting its own energy from light in the environment it is placed in. However, in order to achieve this, it is imperative to design it by using ultra-low power design techniques at all levels of abstraction, while maximizing the energy harvesting and management efficiencies. Several CIS designs with integrated energy harvesting photodiodes have been reported in the past [1,6,7,8,9]. The self-powered image sensor (SPS) concept introduced in [6,7] is based on connecting a floating photodiode in series with the battery supply. Thus, despite its name, the SPS is not really self-powered, but rather generates only a boosted supply voltage. Pixels proposed in [8,9] are based on reconfigurable PN-junctions that could perform both image capture and energy harvesting operations. However, drain junctions of the pixel transistors connected to the anode of energy harvesting photodiode cause significant leakage decreasing the energy harvesting efficiency In pixel transistors receive light and light induced leakage in a PN-junction is much higher than reverse leakage current of an unilluminated junction. The light induced leakage of the reverse biased drain to the substrate junctions of these transistors decreases the energy harvesting efficiency significantly. The energy harvesting and imaging (EHI) pixel structure introduced in [1] is also based on reconfigurable PN-junction photodiodes. As opposed to others, better energy harvesting efficiency was achieved in EHI by decoupling the anode of the energy harvesting photodiode from pixel transistors and other loss paths. However, high power consumption of readout electronics and low energy harvesting capability of the EHI structure made it impossible to attain self-powered operation. The primary focus of these works was to develop a pixel structure with good energy harvesting capability. As a result, none of them addressed low-power electronics and power management circuit design issues for energy autonomous image sensors.

In this paper, the first energy harvesting type CIS design with integrated power management system (PMS) is introduced. The PMS running a low-power maximum power point tracking (MPPT) algorithm was integrated with a dual-mode CIS that contains new, highly efficient EHI pixels and ultra-low power readout electronics. The PMS controls the operating point of energy harvesting photodiodes during energy harvesting, performs DC-DC conversion and voltage regulation, powers the chip with harvested energy when sufficient energy is stored and switches back to battery power when harvested energy is not sufficient.

This article is organized as follows: Section 2 introduces the overall architecture of the proposed EHI imager with PMS. Section 3 explains the design details of the new EHI pixel structure as well as other ultra-low power functional imaging and energy harvesting blocks. Section 4 presents the details of the PMS integrated in the design. Simulated and measured performance characteristics of the EHI CIS are presented in Section 5, while the discussion and conclusions are given in Section 6.

## 2. Architecture Overview 

**Figure 1:**
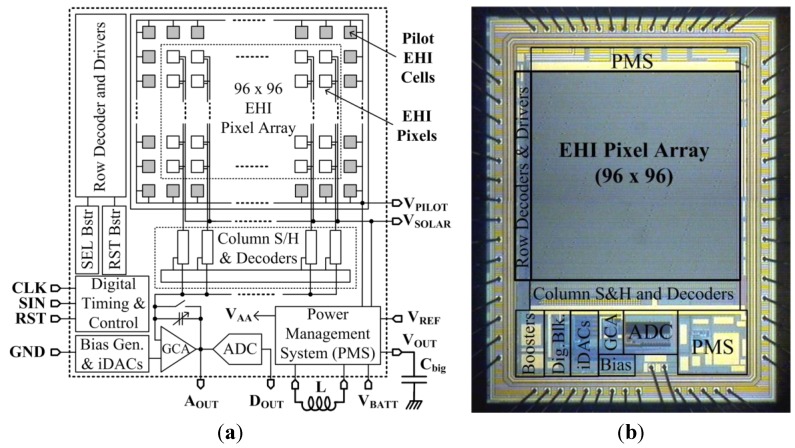
Block diagram and die micrographs of the ultra-low power EHI type CIS.

A block diagram and die micrograph of the new ultra-low power EHI type CIS with 96 × 96 pixel array is shown in Figure 1. The new EHI pixel structure achieves improved energy harvesting efficiency and wider spectral response. The design works in two modes sequentially: (1) imaging mode; and (2) energy harvesting mode.

In *imaging mode (IM)*, the pixel photodiodes work as image capturing devices and convert the light incident on them into voltage. A column series readout architecture with a global charge amplifier and an analog-to-digital converter (ADC) is integrated with a pixel array similar to the EHI imager described in [1]. The pixel array is read out sequentially, like a regular CMOS APS imager with rolling shutter. The pixel output from each row is first sampled by column sample and hold (S & H) circuits. The absolute pixel signal after correlated-double-sampling (CDS) is amplified by a global charge amplifier and converted into digital by a 10-bit successive approximation register (SAR) type ADC. During *energy harvesting mode (EHM)*, pixel photodiodes are configured as solar cells to harvest the solar energy. It is important to harvest the maximum available power from solar cells in this mode. The maximum power point (MPP) of a solar cell changes with illumination level and ambient temperature. Therefore, a maximum power point tracking (MPPT) circuit is integrated in the power management system (PMS) in order to collect energy more efficiently. The PMS monitors the illumination conditions using integrated pilot solar cells, tracks the maximum power point (MPP), and stores the harvested solar energy onto a storage capacitor. Since the solar energy harvester output voltage is less than 0.5 V, an on-chip self-timing boost converter with line regulator is integrated in the PMS to generate the desired supply voltage. The PMS connects the image sensor supply bus to the storage capacitor that holds the harvested energy when sufficient energy is stored. Once the storage capacitor is discharged to a certain level, PMS connects the imager supply back to battery. During EHM all blocks related to imaging are turned off to save power.

Power consumption of the imager is scaled down by using additional low-power circuit design techniques. Supply voltage reduction is an effective tool for reducing the power consumption of both digital and analog circuits. Power consumption in digital circuit blocks is proportional to the square of the supply voltage while in analog blocks it is typically proportional to the supply voltage. Thus, when the supply voltage is reduced from 1.2 V to 1.0 V, the power consumed by digital and analog blocks are reduced approximately 30% and 17%, respectively. Smart use of low-power design techniques for power-hungry blocks such as a global charge amplifier and on-chip ADC without sacrificing performance further reduced the power consumption. Power scheduling in active analog and digital blocks were also adopted. Typically the minimum supply voltage requirement of the pixel electronics is higher than that of other blocks to achieve a good pixel dynamic range. The pixel dynamic range diminishes almost to zero for sub-1 V supply voltages. In the proposed image sensor, global voltage boosters are used for critical nodes in the pixel array to increase the dynamic range of the pixel instead of increasing the supply voltage. As a result, the whole sensor can work under 1-V supply without sacrificing the performance metrics. Additionally, an overridable digital timing and control block is integrated on-chip to reduce the power dissipated by the digital IO pads. The chip was fabricated in a standard 1.8 V, 1P6M, 0.18 µm CMOS process. The total power consumption of the proposed EHI CIS is 6.53 µW on 1 V power supply for a 96 × 96 pixel array while operating at 5 fps frame rates.

## 3. Ultra-Low Power Image Sensor Circuit Design 

Design details of individual imaging mode circuits and the low-power design techniques applied are described in this section.

### 3.1. Energy Harvesting and Imaging (EHI) Pixel

**Figure 2:**
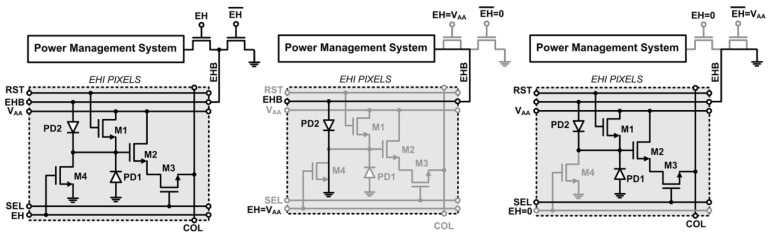
Dual-mode CMOS EHI APS pixel: (**a**) circuit schematic; (**b**) energy harvesting mode (EHM) configuration; (**c**) imaging mode (IM) configuration.

The EHI pixel electronics are identical to the EHI pixel presented in [1] as shown in Figure 2. Transistors M1-M3 are the reset, pixel source follower (PSF) and row select transistors found in a standard three-transistor (3T) CMOS active pixel sensor (APS) pixel. The differences between the EHI and a typical 3T APS pixel are the reconfigurable photodiode (PD2) and the mode select transistor M4. Cathodes of PD1 and PD2 are both connected to the FD node. The anode of PD1 is permanently grounded, while the anode of PD2 is connected to the global energy harvesting bus (EHB). During energy harvesting mode (EHM), the mode select transistor is turned on shorting the FD node to ground. EHB is connected to the power management system (PMS) as shown in Figure 2b. In this configuration, PD1 is shorted and the anodes of the PD2 diodes in all pixels are disconnected from ground. The solar cell array formed by the PD2 diodes delivers the generated energy to the energy harvesting bus (EHB) and the on-chip PMS. PMS controls the load of the solar cell array to harvest the maximum energy from the incident light. In imaging mode (IM), the EHB is connected to ground as shown in Figure 2c. Therefore, both diodes (PD1 and PD2) are reverse biased and work like a typical imaging photodiode. The cathodes of PD1 and PD2 connected to the floating diffusion node (FD node) are reset like a regular 3T APS. The voltage on FD is buffered by the pixel source follower transistor to column sample and hold circuits.

**Figure 3:**
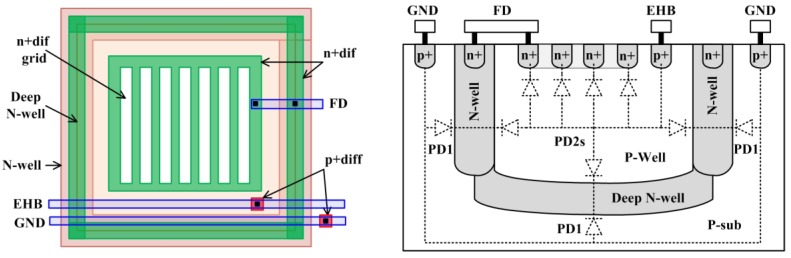
Proposed EHI CMOS APS pixel: (**a**) layout; (**b**) cross section.

The energy harvesting capacity of the imager is increased by using additional layers that exist in the selected CMOS process for building the pixel photodiodes as shown in Figure 3. N-well, deep N-well, isolated P-well, and n+ diffusion (n+dif) layers are used to build the new reconfigurable photodiode PD2 instead of using p+ diffusion (p+dif) and N-well layers as in [1]. Since deep N-well and n+dif layers can be placed on top of each other, two photodiodes are built in the same lateral silicon area. PD2 is composed of a parallel combination of vertically stacked P-well/N-well and P-well/n+dif photodiodes. Therefore, the effective light sensitive area is increased for the same silicon area even though the N-well ring decreases the area available for the P-well and n+dif layers limiting the fill factor for each of the two vertically stacked junctions forming PD2. However, vertical stacking of P-well/N-well and P-sub/N-well junctions results in a larger harvesting pixel fill factor. The pixel pitch in this design is 23 µm and the fill factor for P-well and n+dif regions are 27.5% and 23.1%, respectively. Moreover, the effective junction area for the P-well/deep N-well junction is much larger than the area of a shallow junction with the same lateral silicon area, since the P-well/N-well junction has much deeper sidewalls. Thus, the total junction area is much larger than a p+dif/N-well junction with same lateral silicon area. The total photo-generated current of the proposed EHI structure is considerably higher compared to the reconfigurable EHI photodiode structures presented in [1] since the photo-generated current in a PN-junction is proportional to total junction area [10].

Photons with different wavelengths are absorbed at different depths in silicon. Electron-hole pairs created by the absorbed photon energy recombine unless they are separated by a built-in electric field in the depletion region. Therefore, only photons absorbed in the vicinity of depletion regions contribute to photoelectric processes. Shallow junctions formed between diffusion layers and substrate or wells absorb photons with wavelengths in the green spectrum with higher efficiency. On the other hand, deeper junctions formed between isolated P-well and N-well, and between P-sub and N-well absorb photons with longer wavelengths which could penetrate deeper. Since short wavelength photons (*i.e.*, blue) cannot penetrate into silicon, depletion regions very close to the silicon surface are needed for capturing them with high efficiency. The added junctions in the proposed EHI pixel structure not only increase the energy harvesting capacity, but also improve the spectral response during imaging mode. Since the sidewalls of every junction reach the surface, the total depletion region at the silicon surface depends on the total length of the junction sidewalls. Therefore, dividing the n+dif into grids improves lateral collection regions, [11], and improves the blue sensitivity. Moreover, lightly doped junctions (*i.e.*, P-well/N-well and P-sub/N-well) have wider depletion regions that extend to the surface, further improving the blue sensitivity.

### 3.2. Reset-and-Select Signal Boosters

**Figure 4:**
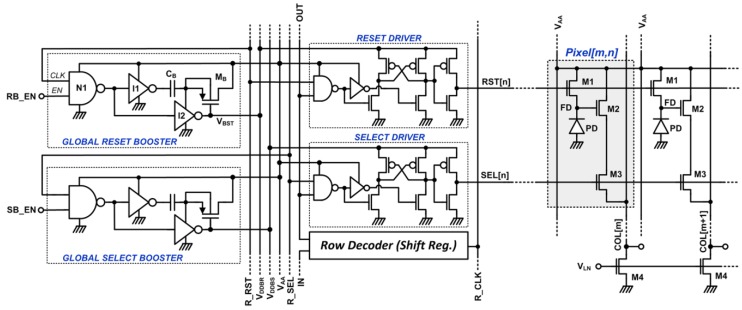
Global, row, and pixel level circuits for reset and select boosting in three-transistor (3T) CMOS APS imagers.

The traditional 3T CMOS APS pixels shown in Figure 4 have serious supply voltage limitations and they are not suitable for low-voltage operation. Gate to source voltage drop across the pixel reset and source follower transistors limit the dynamic range of the pixel and the readout electronics following it. The dynamic range of a pixel is related to the buffering range of the floating diffusion (FD) node voltage by the pixel source follower. Detailed analysis of the input/output range and the improvement ranges for different boosting techniques could be found in [12]. In this design, we adopted the select and reset boosting techniques to extend the dynamic range of the standard 3T APS pixel electronics. Circuit diagram of the global, row, and pixel level circuits that implement reset and select boosting are shown in Figure 4.

The global booster circuit shown in Figure 4 is a single-shot voltage doubler composed of a NAND gate, two inverters, a boosting capacitor, and a PMOS transistor, [1]. During pixel readout, all pixels in a row are reset or selected in parallel. Therefore, reset and select boosters drive the gates of all reset and select transistors on the selected row as well as the parasitic capacitances of the metal interconnects and the row driver circuits shown in the Figure 4. Thus, loads for both reset and select booster circuits are mostly capacitive. As long as no active current is drained from the booster output (V_BST_), high level of the boosted supply voltage for row drivers is given by Equation (1):

(1) $${\text{V}}_{\text{BST}}={\text{V}}_{\text{AA}}+\frac{{\text{C}}_{\text{B}}}{{\text{C}}_{\text{B}}+{\text{C}}_{\text{L}}}{\text{V}}_{\text{AA}}$$

C_L_ in Equation (1) is the load capacitance of the booster. If the boosting capacitance (C_B_) is large enough, good boosting efficiency is achieved and the boosted voltage level is closer to 2V_AA_. When a reset signal with logic high level equal to V_AA_ drives the reset switch, the maximum voltage at the FD node will lower than V_AA_ by the threshold voltage (V_THN_) of the reset transistor. The threshold voltage of the reset transistor will be higher than the nominal threshold voltage due to the body effect. This results in a significant reduction of pixel dynamic range. When the logic high level of the reset signal is larger than V_AA_ + V_THN_, the reset transistor can pull the FD node to V_AA_ with no voltage drop. Boosting the select signal reduces the channel resistance of the select transistor. When the channel resistance is reduced, the voltage drop across the select transistor is reduced. Therefore, boosting the reset and select signals improves the dynamic range, even for lower supply voltages. The use of booster circuits allows reducing the supply voltage from 1.7 V to sub-1 V which leads to further reduction of the total power consumption.

Since only one row of pixels are accessed at a time, one global reset booster and one global select booster circuit are required in the proposed imager. Each row has a row driver and a select driver circuit to drive the reset and select transistors with boosted RST and SEL signals. A cross-coupled high voltage driver was used for this purpose as shown in Figure 4. A dynamic shift register is used as row decoder because of the small pixel array and to reduce the power consumption of the row decoders. The static power consumed by the booster circuits is very low since no current is drawn from the booster circuit outputs. Their dynamic power consumption is also relatively low since they are clocked at very low frequencies.

### 3.3. Readout Circuits

The column series readout architecture in the EHI sensor uses a single global charge amplifier and an ADC for the whole imager. Digital conversion is performed sequentially and data is sent out serially. Using a column series readout architecture in high-resolution and high-speed imagers necessitates prohibitively high-speed ADCs. However, they can easily implemented in imagers designed for low-resolution and low-speed applications. A serial readout architecture is preferred since it consumes a smaller chip area and does not introduce additional fixed pattern noise (FPN) due to mismatch between parallel readout channels [13]. Pixels in the array are read out row by row in a column series architecture. Pixel source followers in the selected row are connected to the column load transistor (M4) through pixel select transistors (M3). Since the source follower output can be pulled towards the input level with unlimited current, the source follower bias current does not have any effect on the sampling time as long as the initial voltage on the column capacitor is lower than the input level. Thus, the column bias current in the proposed imager is set as low as possible reducing the static power consumption of the pixel array.

**Figure 5:**
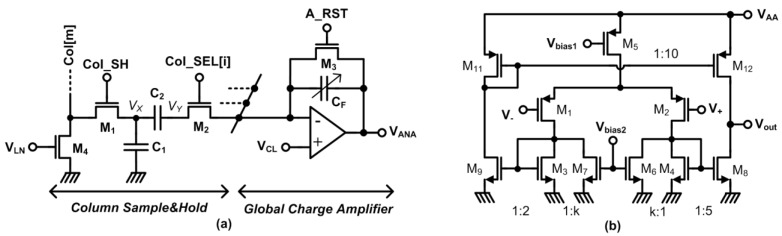
(**a**) Global and column level analog readout circuits; (**b**) current mirror OTA schematic used in global charge amplifier.

Pixel outputs are processed by the column sample and hold (CSH) and global charge amplifier (GCA) circuits as shown in Figure 5. The CSH and GCA effectively function as a switched capacitor programmable gain amplifier. The pixel source follower outputs in the whole row are sampled on CSH capacitors (C_1_, C_2_) at the end of integration (signal level) and right after the pixel reset (reset level) operations. During signal sampling, all column sample-and-hold (M1), column select (M2), and global amplifier reset transistors (M3) are turned on simultaneously. The buffered pixel signal voltage is stored on the CSH capacitor C_2_ against the clamp voltage (V_CL_). Consequently, all pixels in the row are reset. Sample and hold switch (M1) in all columns are turned on again while the column select transistors (M2) are off. The bottom plates of the CSH capacitors (V_x_ side) are charged to the buffered FD reset level and the floating top plate of C_2_ is level shifted by the difference between pixel reset and signal levels. Voltage at the top plate of the sample and hold capacitor C_2_ is given by Equation (2):

(2) $$V_{Y}=V_{CL}+V_{rst}-V_{sig}=V_{CL}+V_{eff}$$

In an integration type image sensor, light incident on the pixel photodiodes is converted to photodiode leakage currents proportional to the light intensity. This light dependent current discharges the pre-charged junction capacitor. The difference between the voltages across the junction capacitor at the beginning and end of the integration period is proportional to the light intensity incident on an individual pixel. If all pixels are reset to exactly the same voltage, the final voltage could be used to extract the light intensity. However, the reset voltage varies pixel-to-pixel due to the threshold voltage, doping concentration, and physical size variations of pixel transistors (M1–M3). Therefore, the final FD node voltage at the end of integration period represents not only the light intensity, but also the fixed pattern noise (FPN) caused by the mismatches. Subtracting the signal level from the reset level for each pixel using the differential sampling scheme described above is known as correlated double sampling (CDS). CDS eliminates not only pixel FPN, but also thermal noise and 1/f noise. True CDS is achieved by subtracting the reset level and signal level for the same integration period. True CDS is not possible in 3T CMOS APS pixels. The CDS described above is a pseudo-CDS where the signal value is subtracted from the reset level of the next integration period [14,15,16,17].

Most of the power in a column readout circuit is consumed during charging and discharging the CSH capacitors. Reducing the CSH capacitor size reduces the power consumption of the imager. Since the columns are read sequentially, the last column is read 1.9 ms after the first column is read in a 96 × 96 resolution imager running at 5 fps frame rate. This delay is longer for lower frame rates. The CSH capacitor discharges due to leakages at V_X_ and V_Y_ nodes during this time. If the capacitor is too small, the voltage drop due to leakage discharge will become significant. Columns that are read out later will appear darker and images will have a brightness gradient. Therefore, capacitor size is picked carefully to achieve a good balance between image quality and power consumption. The leakage increases significantly when semiconductor devices are exposed to light. Thus, special light shields are built above the CSH circuits to protect sensitive column circuits and nodes from light.

### 3.4. Global Charge Amplifier

The global charge amplifier (GCA) shown in Figure 5 performs several critical functions in the analog signal chain. Since the pixel and column readout circuits attenuate the FD signals, GCA restores signal swing and increases the signal to noise ratio (SNR). The GCA also functions as a voltage buffer between the CSH capacitors and the ADC S & H capacitor. Besides, GCA helps CDS operation by forcing the top plates of all CSH capacitors to V_CL_ during signal sampling. Once the differential sampling of the columns is completed, the column select switches are turned on one by one connecting each CSH circuit to the GCA. When the switch is turned on, the amplifier forces *V_y_* in the column readout circuitry to V_CL_. The excess charge on the CSH capacitor C_2_ is moved to feedback capacitor *C_f_*. The amplifier output voltage considering amplifier non-idealities (V_offset_) is given by Equation (3):

(3) $$V_{out}=V_{CL}-\frac{C_{1}C_{2}}{C_{f}\left(C_{1}+C_{2}\right)}V_{eff}+\left(1+\frac{C_{1}C_{2}}{C_{f}\left(C_{1}+C_{2}\right)}\right)\frac{V_{offset}}{1+A_{o}}$$

Designing a very low power charge amplifier is challenging due to the trade-offs between power consumption, settling time and bandwidth. The amplifier has to drive the feedback capacitor, sample and hold capacitor of the on-chip ADC or the analog output pad if analog output is desired. Therefore, the amplifier has to be capable of providing sufficient output current for driving such a large load capacitance. Since the major part of the voltage gain is achieved at the output node, no compensation capacitor is needed in a single stage differential amplifier [16]. Removing the compensation capacitor reduces the power consumption significantly. Balanced current mirror type OTA is a single stage amplifier providing high current driving capability and large output swing. It was shown that the overall power consumption of balanced current mirror OTA is lower than that of the Miller compensated two-stage OPAMP with equal gain, slew rate, gain bandwidth product and phase margin [18].

The circuit diagram of the low-voltage, gain-enhanced current-mirror OTA [19] used in the EHI imager is shown in Figure 5. The additional transistors M6 and M7 reduce the current mirrored to the output branches. Therefore, the output impedance of the amplifier is increased while the transconductance of input transistors remains constant and higher gain is achieved. The gain enhancement ratio is 1/(1−k) where k is the ratio of the currents. Gain enhancement reduces the output current. Since only the output stage current determines the bandwidth, slew rate and settling time, the current mirror ratios are arranged to have an adequate current in the output branch to drive the large capacitive loads, while keeping the current in other branches low as shown on Figure 5. The OTA in the GCA of the proposed EHI imager is designed to drive a 10 pF load while consuming only 2.1 µW and having 1.3 MHz bandwidth. 

### 3.5. 10-Bit SAR ADC

A 10-bit single ended successive approximation register (SAR) type ADC is used for converting the GCA output to a digital code in the EHI imager. SAR ADCs consume relatively low power offering a good balance between chip area and bit resolution. They are suitable for image sensors with medium to high resolutions where ADCs are required to operate at a relatively medium speed. Even though fully differential SAR ADCs have better rejection of common mode noise and even order harmonic distortion, they require doubling the number of switches, two capacitive DACs, more complicated sample and hold circuits and consume more power compared to single ended SAR ADCs. Therefore, single ended SAR ADCs are preferred for low power applications [20,21].

**Figure 6:**
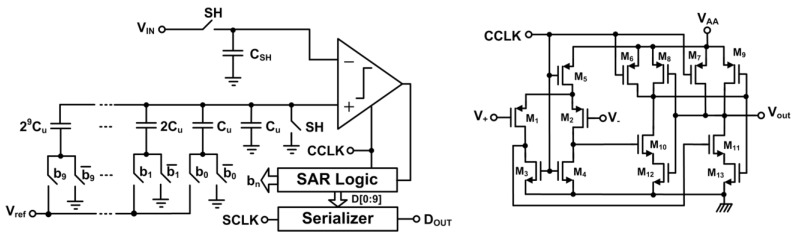
The schematics of the 10-bit SAR ADC and two-stage dynamic comparator.

The schematics of the 10-bit SAR ADC and two-stage dynamic comparator are shown in Figure 6. The ADC uses a 10-bit binary-weighted capacitor array to generate quantization levels. Sampled input voltage (V_in_) is compared with the output of the charge redistributed digital-to-analog converter (DAC). SAR logic implements the binary search algorithm. The direction of the binary search is determined by the comparator output, [22]. During sampling the phase top and bottom plates of all capacitors in the capacitive DAC are connected to ground and GCA output is sampled onto the sample and hold capacitor (C_SH_). After sampling, the bottom plate of the largest capacitor in the DAC is switched to the ADC reference voltage (V_ref_). In this step, the sampled input is compared against V_ref_/2 determining the MSB bit. During the second step, V_in_ is compared against either 3V_ref_/4 or V_ref_/4 depending on the MSB bit determined in the previous step. This process continues until all digital bits are determined and the sampled analog input voltage is converted to the digital domain. Detailed timing diagram and circuit diagrams that implement the binary search algorithm can be found in [23].

The static power consumption of the ADC is ideally zero, if an energy efficient dynamic comparator is used [24]. A true single phase, two-stage comparator is used in the design to reduce the kickback noise. The preamplifier first stage increases the gain for higher sensitivity and faster comparison. The main component of power consumption is the switching power dissipated for charging and discharging the DAC capacitors between GND and V_ref_. Since V_ref_ is set by the output range of the GCA and the clock frequency is determined by the frame rate, the only way to reduce switching power of the SAR ADC is by minimizing the unit capacitor size. Thus, the minimum allowed capacitor size is used to reduce the ADC power consumption. The designed ADC can run at 200 kS/s speed resulting in 20 fps frame rate on 1 V supply. The entire ADC consumes 1.5 µW when running at 50 kS/s corresponding to 5 fps frame rate. 

### 3.6. On-Chip Digital Timing Generator

The EHI chip is a complete camera on a chip system with override capability of all digital control signals through digital I/O pads. All digital control signals for the pixel array, charge amplifier, ADC and the power management block are generated on chip. The size of timing generator is 0.14 mm × 0.14 mm. It consumes 1.485 μW power at 5 fps frame rate. The power consumption of the timing generator is compensated by the reduction of the pad frame power. Since the digital input pads are not necessary, the pad frame consumes significantly less power compared to an imager driven by external clocks. Pads in camera ICs consume more power than non-illuminated ICs due to the light dependent leakage currents of the reverse biased junctions in the large electro static discharge (ESD) protection devices in each pad. The power consumption of the pad frame in the EHI chip while digital input pads are powered up and external signals are applied is 2.64 µW. This consumption is reduced to 531 nW when the digital input pads are powered down.

## 4. Energy Harvesting Circuits

When a photon is absorbed by a semiconductor material, an electron hole pair is generated. The electron and hole eventually recombine unless they are separated. The built-in electric field in the depletion region of a PN-junction drifts electrons from P-region to N-region and holes from N-region to P-region. Thus, photo-generated electrons from holes are separated. Drift of photo-generated minority carriers across the depletion region results in a photo-generated current flowing from the N-region to the P-region of the semiconductor junction. Since the electric field is zero outside of the depletion region, only the carriers generated in the depletion region and those carriers that can reach the depletion region through diffusion are separated by the electric field. Therefore, more carriers can be separated in materials with wider depletion regions and longer diffusion lengths. The depletion region width is wider if lighter doping concentrations are used for the junctions. Excess carriers recombine through several mechanisms, [25,26]. The recombination rate increases with increased doping concentration in all these mechanisms. Carriers diffuse longer distances when the recombination rate is lower. Therefore, diffusion lengths are longer in lightly doped semiconductors. The photo-generated current (I_ph_) is given by Equation (4) [10]:

(4) $$I_{ph}=A\cdot J_{ph}=A\cdot \left(q\cdot G\cdot \left(L_{N}+W_{D}+L_{P}\right)\right)$$

where A is the junction area, G is the carrier generation rate proportional to illumination, W_D_ is the depletion layer width, and L_P_ and L_N_ are the diffusion lengths of holes and electrons, respectively. Since both depletion region width and diffusion lengths are longer in lightly doped semiconductors, the power generation capacity of lightly doped P and N junctions is higher.

Photo-generated carriers flowing across the depletion layer result in a net positive charge in the P-region and a net negative charge in the N-region and the built-in potential of junction is lowered. This change in built-in potential results in a measurable potential difference between the two sides of the junction. When the built in potential of a PN-junction is reduced either by an applied positive bias voltage or excess carrier build up, diffusion of holes from the P-region to the N-region and of electrons from the N-region to the P-region increases. This forward current (I_f_) is the exponential current of the PN-junction independent of light and is only a function of the potential difference between the P and N regions of the photodiode (V_out_). I_f_ and I_ph_ flow in opposite directions and net current flowing out of the anode of a photodiode (I_out_) as a function of photodiode voltage is given by Equation (5) [10]:

(5) Iout=Iph−If=Iph−IS⋅(eVoutnVT−1)

Here n is the diode ideality factor, I_f_ is the forward current, I_ph_ is the photo-generated current, V_out_ is the voltage across the PN-junction, V_T_ is the thermal voltage, and I_s_ is the reverse saturation current of the junction. This equation suggests a first order photodiode model composed of an ideal current source and an ideal diode. Models based on measurements suggest additional shunt and series resistances [27]. However, the deviation from the first order model due to these added parasitic resistances are not significant and relations derived using this first order equation are accurate enough. 

When no external circuit is connected between the terminals of a PN-junction photodiode, no net current flows. The forward current due to the potential barrier lowering and photo-generated current are equal. The potential difference between the terminals in this condition is known as open circuit voltage (V_oc_) and is given in Equation (6):

(6) $$V_{oc}=nV_{T}\cdot \text{ln}(\frac{I_{ph}}{I_{S}}+1)$$

The power output for a specific output voltage (V_out_) is given by Equation (7). V_out_ corresponding to maximum power output (V_MPP_) can be calculated by setting the derivative of Equation (7) to zero. The relation between V_MPP_ and other solar cell parameters is given in Equation (8). Substituting Equation (6) into Equation (8) we get the relation between V_MPP_ and V_oc_ as in Equation (9):

(7) $$P\left(V_{out}\right)=\left(I_{ph}-I_{S}\cdot \left(\exp\left(\frac{V_{out}}{nV_{T}}\right)-1\right)\right)\cdot V_{out}$$

(8) $$V_{MPP}=nV_{T}\cdot \left[\ln\left(\frac{I_{ph}}{I_{S}}+1\right)-\ln\left(1+\frac{V_{MPP}}{nV_{T}}\right)\right]$$

(9) $$V_{oc}=V_{MPP}+nV_{T}\cdot \left[\ln\left(1+\frac{V_{MPP}}{nV_{T}}\right)\right]$$

Even though *V_oc_* and *V_MPP_* does not have a linear relation, the logarithmic curve defined by Equation (9) is quite linear when *V_oc_* changes by a few hundred millivolts. Since both *V_MPP_* and *V_oc_* are logarithmic functions of illumination, large changes in illumination results in small changes in these voltages. Measurements confirm that ratio of *V_oc_* and *V_MPP_* is fairly constant over a wide range of illumination conditions as it is summarized in Equation (10).

(10) $$V_{MPP}=k_{v}\times V_{OC}$$

This equation is the basis of fractional voltage maximum power point tracking (FVMPPT) method [28]. MPPT circuits based on FVMPPT method are very simple to implement. The voltage fraction is determined by measurements under various illumination levels. The MPPT circuit measures the open-circuit voltage (V_oc_) either by using an identical solar cell (pilot cell) or interrupting the solar cell operation and measuring the actual V_oc_. Load of the solar cell is controlled to set the photodiode output voltage equal to the appropriate fraction of open circuit voltage. 

Since the predefined *k_v_* value is just an approximation, FVMPPT circuit is not a true MPPT circuit. The solar cell operating point will miss the MPP point slightly for different illumination levels and temperatures and the solar cell output power might be a little less than the maximum available. However, MPPT circuits themselves consume power. The power consumed by MPPT circuits increases with increased tracking circuit complexity. Naturally, it is desirable to minimize MPPT circuit power consumption, so that it doesn’t reduce the overall power efficiency of the energy harvesting system. The FVMPPT-based MPPT circuits consume very little power compared to complicated true MPPT circuits, so these circuits are the most energy efficient [29].

### 4.1. Integrated Power Management System (PMS)

When the imager is in standby, the EH signal is set to logic high and all imaging mode circuits are powered down. The in-pixel EH switch (M4 in Figure 2a) shorts the anode of permanently grounded photodiode PD1 and the cathode of reconfigurable photodiode PD2 to ground. Anodes of all PD2 photodiodes in 9216 pixels are connected to the energy harvesting bus (EHB). In EH mode the EHB is disconnected from ground and connected to the power management system (PMS). The PD2 photodiodes in all pixels are configured as a micro solar cell array.

**Figure 7:**
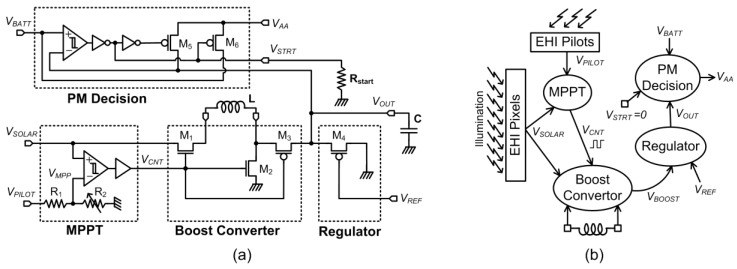
(**a**) Circuit diagram of the power management system (PMS); (**b**) operation principle of the PMS.

The unique integrated power management system (PMS) consists of a maximum power point tracking circuit (MPPT), a boost converter, a voltage regulator, and a power management decision block. PMS circuit and its operation principles are shown in Figure 7.

Solar cells in the array are operated at the maximum power point (MPP) by the maximum power point tracking (MPPT) circuit. The output voltage of the solar cell array is boosted by an inductive boost converter. The output of the boost converter is stored in a large off-chip storage capacitor. The storage capacitor voltage is regulated to a level slightly higher than the battery voltage by using an anti-blooming gate. The power management decision block compares the voltage at the storage capacitor V_OUT_ to the battery voltage V_BATT_. Once V_out_ reaches a sufficient level, the chip internal supply voltage (V_AA_) is switched from battery supply to harvested voltage. In other words, the chip uses the battery voltage for V_AA_ until V_OUT_ is charged to a sufficient level. Once V_OUT_ is charged to this level, the chip is supplied by the harvested energy.

### 4.2. Maximum Power Point Tracker (MPPT) Circuit

Since the power generated on chip is limited, the MPPT circuit has to be as low power as possible. The on-chip MPPT circuit is implemented using a pilot solar cell, a comparator and a 4-bit programmable resistive voltage divider as shown in Figure 7. The open circuit output voltage is generated by a distributed pilot cell structure surrounding the EHI pixel array. The pilot cell is constructed using the same CMOS layers used for building the energy harvesting photodiodes so that pilot cell and pixel energy harvesting photodiodes have the same open circuit voltage. The resistive voltage divider output is the reference voltage (V_MPP_) and *k_v_* is ideally equal to resistive division ratio *k_R_* given in Equation (11):

(11) $$k_{R}=\frac{R_{2}}{R_{1}+R_{2}}$$

Measurements have shown that integrated micro solar cells built in different CMOS processes have a voltage fraction *k_v_* in the range between 0.80 and 0.85. The *k_R_* ratio is controlled by the programmable bottom resistor R_2_ to set *k_v_* to the correct value. Ideally, pilot cells should not be loaded so that pilot cell output voltage (V_PILOT_) is equal to the open circuit voltage. The resistive voltage divider is implemented with a very large on-chip resistor string so that the current drawn from the pilot cell is much smaller than the short circuit current of the pilot cells. Deviation of *V_PILOT_* from *V_oc_* is insignificant for small output currents due the logarithmic dependence of photodiode voltage on the output current. Since the resistive chain is programmable, *k_R_* can be adjusted to include the deviation of *V_PILOT_* from *V_oc_*. The required value for resistive division ratio is given in Equation (12):

(12) $$k_{R}=k_{v}\times \frac{V_{OC}}{V_{PILOT}}$$

The MPPT block comparator continuously monitors whether *V_SOLAR_* is larger than *V_MPP_* and generates a control/clock output (*V_CNT_*) used by the boost converter. The comparator functions as an asynchronous control signal generator for the boost converter. Since the boost converter is the load for the solar cell array, the comparator controls the amount of current sourced from the solar cell array by switching the boost converter. Therefore, it keeps solar cells operating at maximum power point thus optimizing harvesting efficiency.

### 4.3. Boost Converter

When M1 and M2 in the boost converter block are turned on, the solar cell current flows through the off-chip inductor to ground. The output voltage of solar cell (*V_SOLAR_*) drops as the current drawn from the solar cell increases. If *V_SOLAR_* becomes 0 V, the current reaches the maximum available current (short circuit current). MPPT circuit tries to keep the current at the optimum level by sensing the *V_SOLAR_* voltage. When *V_SOLAR_* drops one hysteresis voltage below the *V_MPP_*, the MPPT comparator turns the NMOS switches (M1, M2) off and turns the PMOS switch (M3) on. At this moment, the inductor is floating with one terminal connected to the storage capacitor. Since the inductor current cannot change instantly, the floating inductor will go on supplying a decaying current. Thus, the solar energy stored in the EMF of the inductor is transferred to a large external storage capacitor. Meanwhile, the *V_SOLAR_* voltage rises. When it raises one hysteresis voltage above *V_MPP_*, the output of the MPPT comparator is toggled starting a new cycle. This operation continues indefinitely charging the *V_OUT_* node voltage higher at each cycle with maximum efficiency. The switches in the boost converter are driven by the MPPT circuit. This integrated topology requires no clocks to drive the switches, so the circuit ends up being very simple compared to other switched capacitor charge pump designs. The MPPT algorithm is implemented with no extra power consumption.

### 4.4. Regulator

In order to regulate the harvested voltage output at *V_OUT_* node with low-power consumption, a charge skimming regulation technique was utilized. As the floating inductor (acting like a current source) pumps charge to the storage capacitor, the voltage across the capacitor increases proportionally and decreases as load removes charge from it. If the current consumed from the capacitor is larger than the current supplied, the capacitor will eventually discharge. However, when the supplied current is larger, voltage will go on increasing indefinitely. The charge skimming gate is a simple switch that turns on when the storage capacitor voltage reaches V_REF_+|V_THP_|. When the charge skimming gate turns ON, it will dump the excess charge to ground. As the excess charge is dumped and the voltage level falls, the charge skimming gate will turn OFF. The output voltage might have a small ripple due to transistor switching, but satisfactory performance is achieved using such a simple regulator.

### 4.5. PM Decision Block

The job of the power management (PM) decision block is to switch the chip power supply (*V_AA_*) between the energy storage node (*V_OUT_*) and battery (*V_BATT_*) voltages. It compares the *V_OUT_* and *V_BATT_* with a hysteresis. If *V_OUT_* is one hysteresis voltage (*V_HYST_*) above the *V_BATT_* voltage, then M5 turns on, and M6 turns off allowing self-powered operation. Meanwhile, MPPT and the boost converter continue to transfer charge from the solar cells to the external capacitor keeping *V_OUT_* high. If the harvested and transferred energy is more than the energy consumed by the imager, then regulator transistor M4 turns on and skims the excess charge from the *V_OUT_* node. Here, the regulator turn on voltage could be adjusted above the *V_OUT_*+*V_HYST_* so that more charge is stored on the external capacitor beyond the optimum operating voltage.

**Figure 8:**
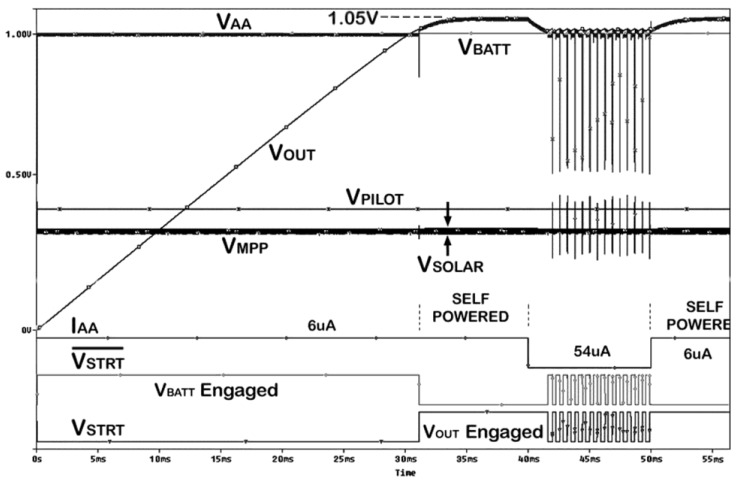
Simulation result of the full operating cycles of the PMS.

A simulation representing full operation cycles of the PMS is shown in Figure 8. In this simulation the battery voltage was set to 1 V, while *k_R_* is 0.8, *V_SOLAR_* is 400 mV and the comparators have 50 mV hysterisis. The storage capacitor charges and the system is self powered when the load current is set to 6 μA. Then it is switched to 54 μA to show the adaptive operation of the PMS under larger loading conditions. The PMS repeatedly switches the supply rail between battery and the harvested energy.

Note that the whole chip including the PMS is powered through the internal *V_AA_* supply line. Thus, *V_AA_* line has to be connected to an auxiliary power source or a battery when the chip is first powered. This is achieved by grounding the gate of M6 through a large off chip resistor (R_START_ > 5 MΩ). The inverter driving the gate of M6 is made stronger accommodating this component. In energy autonomous mode of operation, *V_START_* signal is used as EH signal and imager is switched to energy harvesting mode when *V_OUT_* drops below *V_BATT_* and once *V_OUT_* increases above *V_BATT_*, the imager switches back to imaging mode. Full energy autonomy is possible in this mode. When the incoming light increases, more power is harvested and the imager will stay in energy harvesting mode for a shorter time. As harvested power decreases, the imager will need to harvest energy for a longer time to power the imager for the same amount of time.

## 5. Results and Discussion

The proposed self-powered CMOS image sensor was fabricated in a standard 1.8 V, 1P6M, 0.18 µm CMOS process. The chip occupies a 3 mm × 4 mm silicon area. The image sensor is composed of a 96 × 96 pixel array with a pixel pitch of 23 µm. Compared to other pixel structures the new pixel has two vertically stacked high fill factor photodiodes (PD1 with 83.4%, PD2 with 50.6%) resulting in better photoresponsivity in confined spaces. The energy harvesting capacity of the micro solar cell array composed of the in pixel energy harvesting photodiodes as well as the power output from the boost converter are measured to characterize the power consumption and energy harvesting capacity of the EHI imager. Imaging mode performance characteristics are also provided in this section.

### Imaging Mode Power Consumption 

The general characteristics of the EHI imager are presented in Table 1. Overall power consumption of the EHI imager in imaging mode for 1 FPS, 2.5 FPS, 5 FPS, 10 FPS, and 20 FPS frame rates are provided. Tektronix DMM 4040, 6.5 digit multimeters with 100 pA resolution were used for measuring the current input to the power pads. 

**Table 1:** Performance summary of the EHI imager.

Process	0.18 µm 1P6M CMOS				
Pixel Type	3T CMOS EHI APS				
Pixel Fill Factor (PD1)	83.4%				
Pixel Fill Factor (PD2)	50.6%				
Pixel Array Size	96 × 96				
Supply Voltage (V)	1.0				
ADC Type	SAR				
ADC Resolution	10-bit				
Frame Rate (FPS)	1.0	2.5	5.0	10.0	20.0
Power Consumption (µW)	2.09	3.96	6.53	11.16	19.96

**Table 2:** Performance summary of the EHI imager circuit blocks in imaging mode on 1 V supply.

Frame Rate (FPS)	1.0	2.5	5.0	10.0	20.0
Pixel Array (µW)	0.11	0.12	0.14	0.17	0.23
Row Decoder (µW)	0.06	0.14	0.25	0.50	1.01
Ref. Gen. and Bias Circuits (µW)	0.16	0.16	0.16	0.16	0.16
Column Circuits (µW)	0.010	0.017	0.028	0.050	0.095
Global Charge Amplifier (µW)	0.68	1.40	2.09	2.82	4.22
ADC (µW)	0.31	0.77	1.53	3.07	6.15
Pad Frame (µW)	0.12	0.29	0.53	1.08	2.07
Timing Generator (µW)	0.33	0.77	1.49	3.00	5.72
PM Decision (µW)	0.31	0.31	0.31	0.31	0.31
Total (µW)	2.09	3.96	6.53	11.16	19.96

The imager has separate power supply pads for the pad frame, ADC, power management system, digital block, pixel array and readout electronics. Detailed power consumption of the main circuit blocks running on 1 V supply voltage and different frame rates in imaging mode are given in Table 2. The power management decision block is on during imaging mode so that it can switch the imager power supply back to battery supply when the storage capacitor discharges. Therefore, it is included in the imaging mode power consumption. Global charge amplifier and SAR ADC consume most of the power in the new EHI imager. Power consumption of the amplifier is 2.09 µW with 1 V supply at 5 fps. ADC operates at 50 kS/s conversion speed with 10-bit resolution while consuming only 1.53 µW in this case. ADC can be reconfigured to have 8-bit resolution for lower power consumption instead of 10-bit. At 5 fps frame rate, SAR ADC consumes 1.09 µW power with 8-bit resolution.

**Figure 9:**
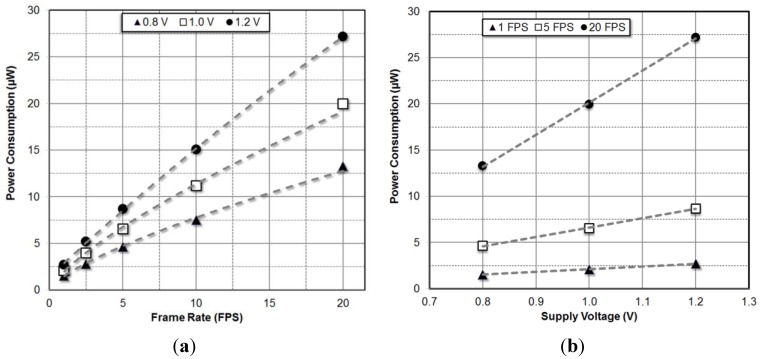
Power consumption of the EHI imager for varying frame rates (**a**) and supply voltages (**b**).

Even though the imager is designed for a nominal operating supply voltage of 1.0 V, it can operate with satisfactory performance down to 0.8 V. Power consumption of the EHI imager in imaging mode for 0.8 V, 1.0 V and 1.2 V supply voltages running at different frame rates are shown on Figure 9.

Power consumption of the digital circuit blocks increases linearly as the operating frequency increases. Power consumption in analog blocks is independent of operating frequency. Since the amplifier output settling time decreases at higher operating frequencies, the amplifier bias current is increased to settle to the final voltage faster. Therefore, even though the charge amplifier is an analog block, its power consumption increases with frame rate. Moreover, the power consumed by the amplifier while charging load capacitances also increases with operating frequency. In practice, the only blocks that consume strictly static power are the reference bias generator, bias circuits and the continuous time comparator in the power management decision block. These blocks consume a small portion of the total chip power. Therefore, power consumption of the EHI imager has an almost linear dependence on the frame rate due to the frame rate adaptive amplifier bias current.

Energy figure of merit (FoM) is an important performance metric for comparing the power consumption of imagers with different resolutions operating at different frame rates [1]. It is defined as the energy consumed for generating the digital output for a single pixel. FoM is given in Equation (13) where P_Total_ is the total power consumption, FR is the frame rate, m is the number of pixel array rows and n is the number of pixel array columns:

(13) $$FoM=\frac{P_{Total}}{FR\cdot m\cdot n}$$

**Figure 10:**
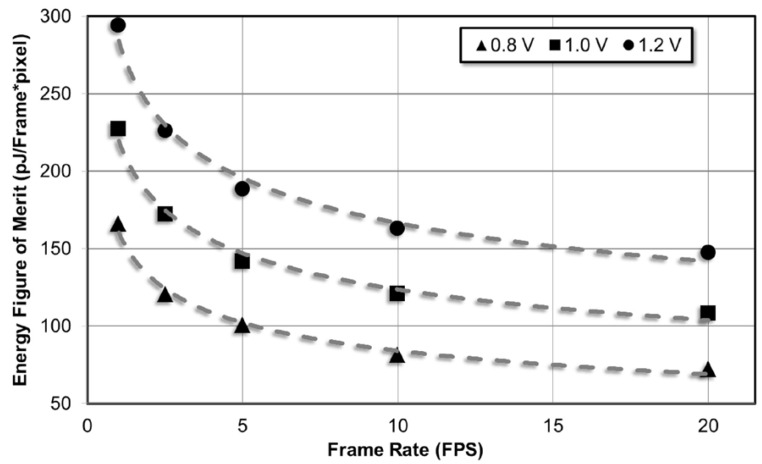
FoM performance of the imager under different frame rate and supply voltages.

Figure 9 also shows the variation of power consumption with supply voltage. The power consumption of digital blocks has a quadratic dependence on supply voltage while the power consumption of analog blocks has an almost linear dependence. Since the power supply rejection of the reference current generator is finite, the bias current increases slightly with increasing supply voltage. Switching power consumption dominates the power consumption at higher operating frequencies, so the power consumption dependence on supply voltage is closer to quadratic for higher operating frequencies. At nominal 1 V supply and 5 fps frame rate, the imager achieves 142 pJ/Frame × pixel efficiency. As expected at 20 fps, this drops to 108 pJ/Frame × pixel. Figure 10 shows the variation of FoM with frame rate for different supply voltages.

**Figure 11:**
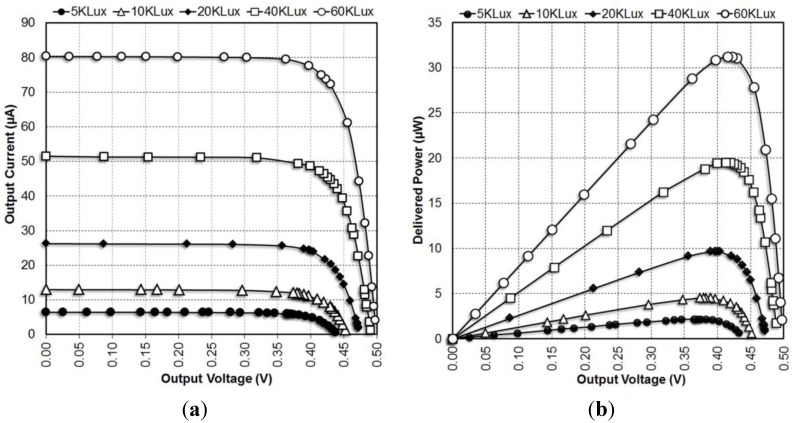
Measured output voltage *vs.* output current (**a**) and output power of the solar cell array (**b**).

The power generated by the on chip solar cell array is measured by turning the boost converter off and directly loading the energy harvesting bus (EHB) with a variable resistor. The current voltage (IV) and power voltage (PV) curves for various illuminations are shown in Figure 11.

Output power from the voltage regulator and the output power from the solar cell array is measured to determine the efficiency of the power management system (PMS) block under 60 Klux illumination conditions. The measurements are performed by varying the reference input to the MPPT comparator to control the solar cell array output voltage. Boost converter efficiency is the ratio of output power from the boost converter to the input power. However, the input power is not constant and it varies for different solar cell voltages. The ratio of output power to the maximum input power is defined as normalized boost converter efficiency. Normalized boost converter efficiency is a better metric for characterizing the boost converter. PMS consumes 2.21 µW total power. This power consumption further reduces the overall efficiency of the power management block.

**Figure 12:**
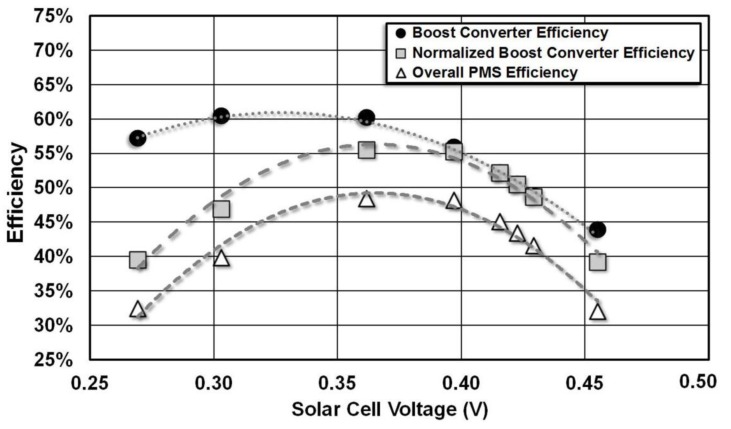
Measured efficiency of boost converter and overall PMS block.

Figure 12 shows how the boost converter efficiency, normalized boost converter efficiency and overall PMS efficiency change for different solar cell voltages. The maximum power point for the solar cell array and the maximum efficiency point for the boost converter do not overlap. The boost converter operates with maximum efficiency of 60.4% when *V_SOLAR_* is 303 mV. The solar cell array delivers the maximum power to the boost converter when *V_SOLAR_* is 422.6 mV. Boost converter delivers maximum output power of 17.32 µW for an input power of 28.8 µW when *V_SOLAR_* is 362 mV. This input power is less than the maximum output power of 31.2 µW available from the solar cell array. The maximum overall efficiency of PMS is measured to be 48.4%. These measurements suggest operating the MPPT comparator at this voltage instead of the maximum power point of the solar cell array increases the power delivered by the PMS. These results are summarized in Table 3.

**Table 3:** Performance summary in energy harvesting mode.

PM Decision Power (µW)	0.31
MPPT+Boost Converter Power (µW)	1.90
Total PMS Power Consumption (µW)	2.21
Boost Converter Efficiency	60.4%
Normalized Boost Converter Efficiency	55.5%
Overall PMS Efficiency	48.4%

Power consumption and energy harvesting capacity of the proposed imager with the other energy harvesting image sensors is presented in Table 4. The power harvesting capacity of the proposed imager is significantly higher than other imagers and energy figure of merit is second after [9].

**Figure 13:**
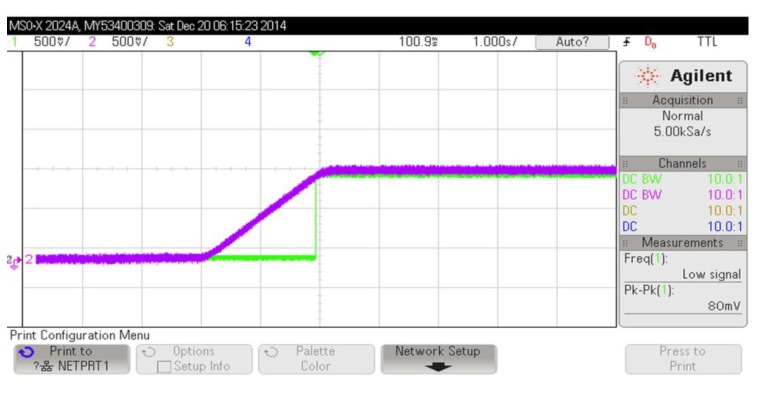
Measured storage capacitor voltage (V_out_) and V_START_ signals.

The *V_START_* signal goes high when the output capacitor is charged and the regulated voltage is more than the battery supply as shown in Figure 13 indicating the system is self-powered. The power output from the PMS block is almost three times the power consumed at 5 fps frame rate. This allows the imager to operate with 72.5% duty cycle. This means that the imager can run for 72.5 s autonomously while disconnected from the battery using the power harvested for 27.5 s.

**Table 4:** Comparison of proposed imager to other energy harvesting imagers.

PARAMETER	[1] Ay	[8] Law	[9] Tang	This Work	
Technology	0.5 µm/5 V (2P3M)	0.35 µm (2P3M)	0.35 µm (2P3M)	0.18 µm/1.8 V (1P6M)	
Pixel Pitch	21 µm	15 µm	10 µm	23 µm	
Array Size	54 × 50	32 × 32	128 × 96	96 × 96	
Fill Factor	PD1-62%/PD2-32%	21.0%	39%	PD1-83.4%/PD2-50.6%	
ADC Resolution	10 bit SAR	8 bit-Ramp	10 bit-Ramp	10 bit SAR	
Supply Voltage	1.2 V	1.5 V	1.35 V	1.0 V	
Energy Harvesting (µW)	2.10 @ 20 klx/ 3.35 @ 60 klx	0.0356 @ 29 klx	3.7 @ 35 klx *	31.2 @ 60 klx/19.5 @ 40 klx	
Energy Harvesting (µW/mm^2^)	1.76 @ 20 klx/ 2.81 @ 60 klx	0.154 @ 29 klx	0.68 @35 klx	6.4 @ 60 klx/4.0 @ 40 klx	
Frame Rate (FPS)	7.4	21	9.6	1	20
Power Consumption (Whole Chip) (µW)	14.25	15.8	10	2.09	19.96
Power Consumption (Pixel Array) (µW)	0.0264	NA	0.37	0.11	0.23
eFOM (Whole Chip) (pJ/frame * pixel)	713.2	765	84	226.8	108.3
eFOM (Pixel Array) (pJ/frame * pixel)	1.32	NA	3.2	11.9	1.25

* Calculated using reported open circuit voltage and short circuit current and assuming 0.76% power fill factor.

## 6. Conclusions/Outlook

An ultra-low power EHI-type CMOS APS image sensor capable of energy harvesting with an on-chip power management system (PMS) is presented. The new energy harvesting photodiodes built with P-well layer increase the power harvesting capacity significantly compared to the energy harvesting photodiodes built with p-diffusion layer reported in other works. The proposed EHI system is capable of harvesting more power than it consumes under normal indoor lighting conditions. Integrated power management system turns the imager on whenever sufficient energy is stored on the storage capacitor and puts it in standby mode when the storage capacitor discharges. The power management system is capable of automatically switching the imager power supply bus between battery and harvested power with 72.5% duty cycle. 

This self-powered structure has great potential for applications requiring extended operation lifetimes with a limited battery supply without the need for any human interference and sufficient light is available. A promising application is a retinal implant. Once the sensor is implanted in the eye, battery replacement is not an option. However, plenty of light is available. Therefore, an image sensor which can use the available ambient light as an energy source is a promising solution for this application.
